# Quantum transport in a single molecular transistor at finite temperature

**DOI:** 10.1038/s41598-021-89436-5

**Published:** 2021-05-17

**Authors:** Manasa Kalla, Narasimha Raju Chebrolu, Ashok Chatterjee

**Affiliations:** 1 School of Physics, University of Hyderabad, Hyderabad, 500046 India; 2 Department of Physics, Central University of Karnataka, Kalaburagi Dist., 585 367 India

**Keywords:** Nanoscience and technology, Physics

## Abstract

We study quantum transport in a single molecular transistor in which the central region consists of a single-level quantum dot and is connected to two metallic leads that act as a source and a drain respectively. The quantum dot is considered to be under the influence of electron–electron and electron–phonon interactions. The central region is placed on an insulating substrate that acts as a heat reservoir that interacts with the quantum dot phonon giving rise to a damping effect to the quantum dot. The electron–phonon interaction is decoupled by applying a canonical transformation and then the spectral density of the quantum dot is calculated from the resultant Hamiltonian by using Keldysh Green function technique. We also calculate the tunneling current density and differential conductance to study the effect of quantum dissipation, electron correlation and the lattice effects on quantum transport in a single molecular transistor at finite temperature.

## Introduction

With spectacular advances in the development of nano-fabrication techniques, recent years have witnessed unprecedented upsurge in research activity in the field of nano-electronics. In this context, the subject of moletronics has received particular attention. Aviram and Ratner^1^ were the first to fabricate a molecular electronic device. They constructed, in 1974, a molecular rectifier using a single organic molecule. On the other hand, a single molecular transistor (SMT) appears to have been first devised in 2000 by Park et al.^2^ who observed nano-mechanical oscillations in a single-$C_{60}$ transistor. An SMT device normally consists of an arrangement in which a molecule or a quantum dot (QD) or any nanosystem constitutes the central part which is connected by metallic leads to a source one side and drain on the other. One of the important reasons for considering a QD or a nanosystem at the central part of an SMT device is that these systems have discrete energy levels. Tuning the gate voltage one can control the current through an SMT device^3–5^. Research on single molecular transistors (SMTs)^6–9^ has lately grown by leaps and bounds, for these devices hold immense promise in technological applications at the nanoscale. Indeed they can be used as spin filters^10^, switching devices^11^, sensors^12^ and so on. Furthermore, an SMT device exhibits many important correlation effects like Kondo effect^13–16^ due to magnetic impurity, Coulomb blockade^17^ due to electron–electron (e–e) interaction and polaronic effects due to electron–phonon (e–p) interaction. They can also have interesting non-equilibrium properties. Many research groups have theoretically studied using different techniques the phonon-assisted transport properties^18–24^ of SMT devices with a single molecule or a quantum dot (QD) as the central molecule.

Chen et al.^25^ have considered e–p interaction in an SMT system and showed how the polaronic effects modify the transport properties. They have used the non-equilibrium Green function technique due to Keldysh and determined the spectral density, tunneling current and differential conductance. They have shown that e–p interaction gives rise to phonon side bands in the spectral density function. The e–p interaction has also been shown to reduce the current density and differential conductance. In a recent work, Raju and Chatterjee^26^ have investigated the effect of quantum dissipation on the transport properties of an SMT device employing non-equilibrium Green function technique of Keldysh. To incorporate the damping effect arising from the interaction of the local QD phonon with the phonons of the substrate, they have added the Caldeira–Leggett term to the Anderson–Holstein Hamiltonian and called it the Anderson–Holstein–Caldeira–Leggett (AHCL) model. As expected, they have also demonstrated a reduction in the current density as well as the differential conductance due to the polaronic effect. Interestingly, however, the damping effect induced by the substrate has been shown to enhance the tunneling current. In a more recent work^27^, the present authors have investigated the role of a magnetic field on the non-equilibrium transport in an SMT device in the presence of electron correlation, polaronic interaction and quantum dissipation due to the interaction between the QD phonon and substrate oscillator modes. Besides the spectral density, current density and differential conductance, we have also calculated the spin polarization. We have shown that the degeneracy in the electron energy due to the spin degree of freedom is removed in the presence of the external magnetic field and the device can be used as a spin filter.

The temperature dependence of the transport properties of three terminal devices like single molecular transistor has been studied by several investigators^28–30^ in recent years. However, the theoretical study has been few and far between^31^. In the present paper we make an attempt in this direction. We consider an SMT device in which the central QD is assumed to have the onsite electron correlation and the Holstein e–p interaction and use the AHCL model to investigate the effect of temperature and quantum dissipation on the non-equilibrium quantum transport in an SMT device employing the finite-temperature Green function technique of Keldysh.

## Model

**Figure 1:**
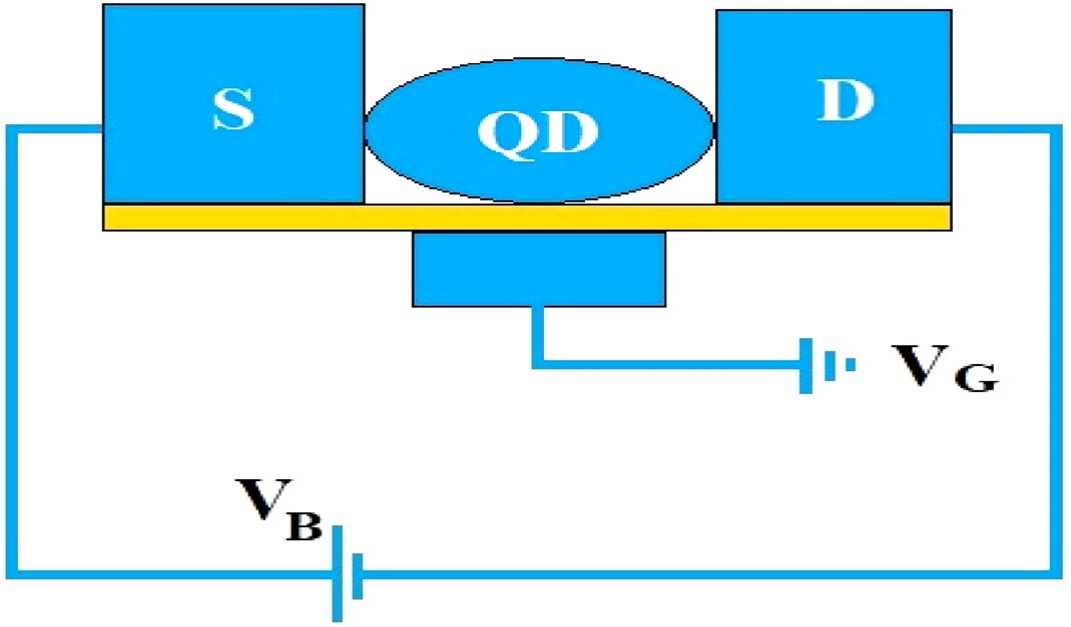
Schematic diagram of an SMT device showing a QD placed on a substrate and connected to source S and drain D. *V*_*B*_ is the bias voltage and *V*_*G*_ is the gate voltage.

Figure 1 describes an SMT device where QD refers to the central QD and $S$ and $D$ represent the conducting leads that play the roles of the source and the drain respectively. The QD molecule is considered to have a single vibrational mode that interacts with the charge carrier in QD through the e–p interaction. The system of QD flanked by S and D is placed on an insulating substrate that plays the role of a heat reservoir. $V_{B}$ and $V_{G}$ are the bias voltage and the gate voltage respectively. The interaction between the local QD phonon and the substrate phonon modes is considered linear following the Caldeira–Leggett model. This interaction gives rise to a damping effect on the dynamics of the QD phonon. The Hamiltonian of the system is thus modeled by,

1 $$H = H_{l} + H_{QD} + H_{t} + H_{B} .$$

In the above equation, $H_{l}$ represents the Hamiltonian for the source ($l =$ S) and the drain ($l =$ D) and is given by: $H_{l} = \mathop \sum \limits_{k\sigma \in S,D} \varepsilon_{k} n_{k\sigma }$, where $n_{k\sigma } \left( { = c_{k\sigma }^{\dag } c_{k\sigma } } \right)$ denotes the number operator for conduction electrons with wave vector ${\varvec{k}}$ and spin $\sigma$ in the continuum states of S and D, $c_{{{\varvec{k}}\sigma }}^{\dag } \left( {c_{{{\varvec{k}}\sigma }} } \right)$ representing the creation (annihilation) operator of the conduction electrons in the leads. $H_{QD}$ describes the central QD and is modeled as

2 $$H_{{{\text{QD}}}} = \mathop \sum \limits_{\sigma } (\varepsilon_{d} - eV_{g} )n_{d\sigma } + Un_{d,\sigma } n_{d, - \sigma } + \hbar \omega_{0} b^{\dag } b + \lambda \hbar \omega_{0} \left( {b^{\dag } + b} \right)\mathop \sum \limits_{\sigma } n_{d\sigma } ,$$

where $n_{d\sigma } \left( { = c_{d\sigma }^{\dag } c_{d\sigma } } \right)$ refers to the number operator for the localized electrons in the central QD which is assumed to have a single localized level of energy $\varepsilon_{d}$, $c_{d\sigma }^{\dag } (c_{d\sigma } )$ representing the corresponding electron creation (annihilation) operator, $U$ denotes the onsite repulsive coulomb correlation energy, $b^{\dag } \left( b \right)$ stands for the creation (annihilation) operator for the localized QD phonon mode of wave-vector-independent frequency $\omega_{0}$ and $\lambda$ is the onsite e–p interaction strength in QD. Throughout our calculation, we shall consider $\hbar = 1$ for the sake of simplicity. The Hamiltonian $H_{t}$ represents tunneling between the leads and the QD and is given by: $H_{t} = \mathop \sum \limits_{k\alpha \varepsilon S,D} \left( {V_{k} c_{k\alpha }^{\dag } c_{d\sigma } + h.c} \right),$ where $V_{k}$ denotes the hybridization strength between the QD and the source or the drain. The substrate phonons can be described by the Hamiltonian: $H_{BO} = \mathop \sum \nolimits_{j = 1}^{N} \left[ {\left( {p_{j}^{2} /2m} \right) + (m_{j} \omega_{j}^{2} x_{j}^{2 } /2)} \right],$ where $x_{j}$ and $p_{j}$ are respectively the position coordinate and the momentum of the *j*-th oscillator of mass $m_{j}$ and frequency $\omega_{j}$. The coupling of the QD phonon with the bath oscillators is taken as: $H_{vib - B} = \mathop \sum \limits_{j = 1}^{N} \beta_{j} x_{j} x_{0} ,$ following the Caldeira–Leggett model^32^, where $x_{0}$ is the position coordinate of the QD oscillator, $x_{j}$ is that of *j*-th bath oscillator and $\beta_{j}$ is the corresponding coupling strength between the two. The spectral function of the bath phonons can be written as: $J\left( \omega \right) = \mathop \sum \limits_{j = 1}^{N} \left( {\beta_{j}^{2} /2m_{j} \omega_{j} } \right)\delta \left( {\omega - \omega_{j} } \right).$ The Hamiltonian corresponding to the bath and the bath-QD-phonon interaction is thus given by: $H_{B} = H_{BO} + H_{vib - B}$.

## Formulation

### Decoupling of the bath phonons

The QD-phonon-bath interaction can be eliminated exactly by a canonical transformation which essentially renormalizes the phonon frequency $\omega_{0}$ of QD to $\tilde{\omega }_{0}$ which is given by: $\tilde{\omega }_{0} = \left( {\omega_{0}^{2} - \Delta \omega^{2} } \right)^{1/2}$, where $\Delta \omega^{2} ( = \mathop \sum \limits_{j = 1}^{N} \left[ {\beta_{j}^{2} /\left( {m_{0} m_{j } \omega_{j}^{2} } \right)} \right]^{1/2} )$ is the change in $\omega_{0}^{2}$ driven by the interaction with the bath phonons. The vibrational part of the total SMT Hamiltonian reduces to

3 $$H_{vib} + H_{B} = \left( {\frac{{p_{0}^{2} }}{{2m_{0} }} + \frac{1}{2} m_{0} \tilde{\omega }_{0}^{2} x_{0}^{2} } \right) + \mathop \sum \limits_{j = 1}^{N} \left( {\frac{{\tilde{p}_{j}^{2} }}{{2m_{j} }} + \frac{1}{2} m_{j} \omega_{j}^{2} \tilde{x}_{j}^{2} } \right),$$

where $\tilde{x}_{j} = \left[ {x_{j} + \beta_{j} x_{0} /\left( {m_{j} \omega_{j}^{2} } \right)} \right], \tilde{p}_{j} = - i\hbar \left( {\partial /\partial \tilde{x}_{j} } \right).$ Equation (4) shows that the QD and the substrate phonons are now decoupled. If $N$ is very large, the *j*-summation in the expression of $\Delta \omega^{2}$ can be replaced by an integration over $\omega$ and then $\Delta \omega^{2}$ can be written as: $\Delta \omega^{2} = 2\mathop \smallint \limits_{0}^{\infty } \left( {J\left( \omega \right)/m_{0} \omega } \right)d\omega ,$ where $J\left( \omega \right)$ is the spectral density.

In the strict Ohmic case, the spectral density $J\left( \omega \right)$ obeys the relation: $J\left( \omega \right) = 2m_{0} \gamma \omega$ for all frequencies $\omega$, where γ which is called the Ohmic damping coefficient can be written as: $\gamma = \mathop \sum \nolimits_{j = 1}^{N} \left( {\beta_{j}^{2} /2m_{j} \omega_{j}^{2} } \right)\delta \left( {\omega - \omega_{j} } \right)$. The form of the pure Ohmic spectral density obtained above is however not very realistic in the sense that it diverges in the limit of $\omega \to \infty$. One therefore introduces a cut-off frequency to salvage the situation. Various forms have been suggested in this context. We use the Lorentz–Drude form^33^ according to which $J\left( \omega \right)$ is given by:

4 $$J\left( \omega \right) = \frac{{2m_{0} \gamma \omega }}{{\left[ {1 + \left( {\frac{\omega }{{\omega_{c} }} } \right)^{2} } \right]}},$$

where $\omega_{c}$ is the cutoff frequency which is much greater than the other characteristic frequencies of the SMT system. One can immediately see that in the limit $\omega \to \infty$, $J\left( \omega \right)$ goes to zero and in the small-$\omega$ limit, one gets back the pure Ohmic spectral density. The change in the QD phonon frequency can be finally written as:

$$\Delta \omega^{2} = \frac{2}{{m_{0} }}\mathop \smallint \limits_{0}^{\infty } \frac{J\left( \omega \right)}{\omega }d\omega = \gamma \mathop \smallint \limits_{0}^{\infty } \frac{d\omega }{{\left[ {1 + \left( {\frac{\omega }{{\omega_{c} }} } \right)^{2} } \right]}} = 2\pi \gamma \omega_{c} .$$

### Elimination of e–p interaction

Next we eliminate the e–p interaction applying the Lang–Firsov transformation^34^ to the transformed QD Hamiltonian $\overline{H}$ with the unitary operator: $U = e^{s} , S = \lambda \left( {b^{\dag } - b} \right)\mathop \sum \limits_{\sigma } n_{d\sigma } .$ The total effective Hamiltonian of the SMT system becomes

5 $$\tilde{H} = e^{s} \overline{H}e^{ - s} = \mathop \sum \limits_{k\alpha \varepsilon S,D} \varepsilon_{k} n_{k\alpha } + \mathop \sum \limits_{\sigma } \tilde{\varepsilon }_{d\sigma } n_{d\sigma } + \tilde{U}n_{d,\sigma } n_{d, - \sigma } + \hbar \tilde{\omega }_{0} b^{\dag } b + \mathop \sum \limits_{k\alpha \varepsilon S,D} \left( {\tilde{V}_{k} c_{k\alpha }^{\dag } c_{d\sigma } + h.c} \right),$$

where $\tilde{\varepsilon }_{d\sigma } \left( { = \varepsilon_{d} - eV_{g} - \lambda^{2} \hbar \tilde{\omega }_{0} } \right)$ is the phonon-induced modified energy of the QD system, $\tilde{U}$ $\left( { = U - 2\lambda^{2} \hbar \tilde{\omega }_{0} } \right)$ is the renormalized online Coulomb interaction strength and $\tilde{V}_{k}$ ($= V_{k} e^{{ - \lambda \left( {b^{\dag } - b} \right)}} )$ is the effective tunneling strength. $\tilde{\varepsilon }_{d\sigma } , \tilde{U}$ and $\tilde{V}_{k}$ are given by.

### Tunneling current and spectral function: the Keldysh method

The tunneling current ^35–38^ passing through QD is given by

6 $$J = \frac{e}{2h}\smallint \left( {\left\{ {f_{s} \Gamma_{s} - f_{D} \Gamma_{D} } \right\}{\text{A}}\left( \omega \right) + \left( {\Gamma_{S} - \Gamma_{D} } \right)G^{ < } \left( \omega \right)} \right)d\omega ,$$

where $${\Gamma }_{S\left( D \right)}$$ is the coupling strength between QD and the source (drain) and is given by: $${\Gamma }_{S\left( D \right)} \left( {\varepsilon_{k} } \right) = 2\pi \rho_{S\left( D \right)} \left( {\varepsilon_{k} } \right)\overline{{\tilde{V}}}_{k} V_{k}^{*} ,$$ $$\overline{{\tilde{V}}}_{k}$$ being the average of $$\tilde{V}_{k}$$ with respect to the *n*-phonon state and $$\rho_{S\left( D \right)} ,$$ the density of states in the S(D) channel, $$f_{S\left( D \right)} \left( \varepsilon \right)$$ is the Fermi distributions for S(D) and is given by: $$f_{S\left( D \right)} \left( \varepsilon \right) = \left[ {e^{{\left( {\mu_{S\left( D \right)} - \varepsilon } \right)/k_{B} T}} + 1} \right]^{ - 1}$$, $$\mu_{S,D}$$ being the corresponding chemical potentials which are related to $$V_{B}$$ as: $$eV_{b} = \left( {\mu_{S} - \mu_{D} } \right).$$ It is also useful to define a mid-voltage ($$V_{m} )$$ such that the energy level corresponding to it would lie in the middle of the source and drain chemical potential energies. Thus, $$eV_{m} = \left( {\mu_{S} + \mu_{D} } \right)/2.$$ We can assume that $$\mu_{D}$$ is approximately equal to the Fermi energy ($$\varepsilon_{F}$$) corresponding to the drain, then the chemical potential of the source will be given by:$$\mu_{S} = eV_{b} + \varepsilon_{F} .$$ Then, we can write: $$eV_{m} = \frac{1}{2}eV_{b} + \varepsilon_{F} .$$ $${\text{A}}\left( \omega \right)$$ is the spectral function which describes the possible excitation energy spectrum and is given by:$${\text{A}}\left( \omega \right) = i\left[ {G_{dd}^{r} \left( \omega \right) - G_{dd}^{a} \left( \omega \right)} \right] = i\left[ {G_{dd}^{ > } \left( \omega \right) - G_{dd}^{ < } \left( \omega \right)} \right]$$, $$G_{dd}^{r\left( a \right)} \left( \omega \right)$$ being the retarded (advanced) Green function and $$G_{dd}^{ < ( > )} \left( \omega \right)$$ the energy-dependent lesser (greater) Keldysh Green function for the QD electron. These Green functions can be obtained by performing Fourier transformations of $$G_{dd}^{r\left( a \right)} \left( {\tau = t - t^{\prime}} \right)$$ and $$G_{dd}^{ < ( > )} \left( {\tau = t - t^{\prime}} \right)$$ which are given by

7 $$G_{dd}^{r\left( a \right)} \left( {t - t^{\prime } } \right) = \mp i \theta \left( { \pm t \mp t^{\prime } } \right)0\left| {\left\{ {\tilde{c}_{d} \left( t \right),\tilde{c}_{d}^{\dag } \left( {t^{\prime } } \right)} \right\}} \right|0,$$

8 $$G_{dd}^{ < } \left( {t - t^{\prime } } \right) = i\left\langle 0 \right|\tilde{c}_{d}^{\dag } \left( {t^{\prime } } \right)\tilde{c}_{d} \left( t \right)\left. {|0} \right\rangle , \quad G_{dd}^{ > } \left( {t - t^{\prime } } \right) = i\left\langle 0 \right|\tilde{c}_{d} \left( t \right) \tilde{c}_{d}^{\dag } \left( {t^{\prime } } \right)\left. {|0} \right\rangle ,$$

with $c_{d\sigma } \left( t \right) = e^{{ - i\tilde{H}_{el} t}} c_{d\sigma } e^{{i\tilde{H}_{el} t}} , \tilde{c}_{d\sigma } \left( t \right) = \hat{O}c_{d\sigma } ,$ and $\left. {\left| 0 \right.} \right\rangle$ represents the true ground state of the whole SMT system i.e.,$\left. {|0} \right\rangle = \left. {|0} \right\rangle_{el} \otimes \left. {|0} \right\rangle_{ph}$ and $\hat{O}{ } = e^{{ - \lambda \left( {b^{\dag } - b} \right)}} .$ We determine the average value of occupancy on QD employing the expression:$n_{d\sigma } = \smallint d\omega \left[ {\left( {f_{s} {\Gamma }_{s} + f_{D} {\Gamma }_{D} } \right){\text{A}}\left( \omega \right)/2\pi {\Gamma }} \right]$. For the sake of simplicity, we assume the coupling between the central QD and the leads to be symmetric. We can then write:${\Gamma }\left( \omega \right) = { }\left( {{\Gamma }_{S} \left( \omega \right) + {\Gamma }_{D} \left( \omega \right)} \right)/2$, where we invoke the approximation in which we replace ${\Gamma }_{S\left( D \right)}$ by its expectation value with respect to the n-phonon state. ${\Gamma }_{S\left( D \right)}$ is given by ${\Gamma }_{S\left( D \right)} = {\Gamma }_{0} \exp \left[ { - \lambda^{2} { }\left( {f_{ph} + 1/2} \right)} \right]$, where ${\Gamma }_{0} = 2\pi \rho \left( 0 \right)\left| {V_{k} } \right|^{2}$ and $f_{ph} = \left[ {exp\left( {\hbar \tilde{\omega }_{0} /k_{B} T} \right) - 1} \right]^{ - 1}$ is the phonon distribution at temperature $T$. To obtain the spectral function, we decouple the Keldysh Green functions into electron and phonon parts in the limit of weak molecule-lead coupling i.e.${\Gamma }_{S\left( D \right)} \ll \hbar \omega_{0}$. This is equivalent to the Born–Oppenheimer approximation. Thus we can write

9a $$G_{dd}^{ < } \left( \tau \right) = i\left\langle {0{|}} \right.c_{d}^{\dag } \left( 0 \right)c_{d} \left( \tau \right)\left. {{|}0} \right\rangle_{el} \left\langle {\hat{O}^{\dag } \left( 0 \right)\widehat{O }\left( \tau \right)} \right\rangle_{ph} = \tilde{G}_{dd}^{ < } \left( \tau \right)\left\langle {\hat{O}^{\dag } \left( 0 \right)\hat{O}\left( \tau \right)} \right\rangle_{ph}$$

9b $$G_{dd}^{ > } \left( \tau \right) = - i\left\langle {0{|}c_{d} \left( 0 \right)c_{d}^{\dag } \left( \tau \right){|}0} \right\rangle_{el} \left\langle {\hat{O}^{\dag } \left( 0 \right)\widehat{O }\left( \tau \right)} \right\rangle_{ph} = \tilde{G}_{dd}^{ < } \left( \tau \right)\left\langle {\hat{O}^{\dag } \left( 0 \right)\hat{O}\left( \tau \right)} \right\rangle_{ph}$$

$\left\langle {\hat{O}^{\dag } \left( 0 \right)\hat{O}\left( \tau \right)} \right\rangle_{ph}$ is the phonon average with respect to the n-phonon state

10 $$\left\langle {\hat{O}^{\dag } \left( 0 \right)\hat{O}\left( \tau \right)} \right\rangle_{ph} = \frac{{\mathop \sum \nolimits_{n = 0}^{\infty } \left\langle {n\left| {e^{{ - \beta \tilde{H}_{ph} }} \hat{O}^{\dag } \left( 0 \right)\hat{O}\left( \tau \right)} \right|n} \right\rangle }}{{\mathop \sum \nolimits_{n = 0}^{\infty } \left\langle {n\left| {e^{{ - \beta \tilde{H}_{ph} }} } \right|n} \right\rangle }} = e^{{ - \varphi \left( { - \tau } \right)}} = \mathop \sum \limits_{n = - \infty }^{\infty } L_{n} e^{{in\tilde{\omega }_{0} \tau }}$$

where $\tilde{H}_{ph} = \hbar \tilde{\omega }_{0} b^{\dag } b$. Thus we have

11 $$G_{dd}^{ < } \left( \tau \right) = \tilde{G}_{dd}^{ < } \left( \tau \right)\mathop \sum \limits_{n = - \infty }^{\infty } L_{n} e^{{in\tilde{\omega }_{0} \tau }} ,\quad G_{dd}^{ > } \left( \tau \right) = \tilde{G}_{dd}^{ > } \left( \tau \right)\mathop \sum \limits_{n = - \infty }^{\infty } L_{n} e^{{in\tilde{\omega }_{0} \tau }} ,$$

where

12 $$\varphi \left( { \mp \tau } \right) = \lambda^{2} \left[ {\left( {2f_{ph} + 1} \right) \mp \left[ {f_{ph} \left( {1 + f_{ph} } \right)} \right]^{1/2} 2cos\left( {\hbar \tilde{\omega }_{0} \left( { \mp \tau + i/2k_{B} T} \right)} \right)} \right].$$

13 $$L_{ \pm n} = \exp \left[ { - \lambda^{2} \left( {2f_{ph} + 1} \right) + \left( {n\tilde{\omega }_{0} /2k_{B} T} \right)} \right]I_{n} .$$

where $I_{n}$ is Modified Bessel function of second kind and $L_{ \pm n}$ is the spectral weights of the $+ n$th and − *n*th phonon.

side bands as mentioned in^25^. Fourier transform of Eq. (9) gives the spectral function of SMT as

14 $${\text{A}}\left( \omega \right) = \mathop \sum \limits_{n = - \infty }^{\infty } iL_{n} \left( z \right)\left[ {\tilde{G}^{ > } \left( {\omega - n\tilde{\omega }_{0} } \right) - \tilde{G}^{ < } \left( {\omega + n\tilde{\omega }_{0} } \right)} \right] = \mathop \sum \limits_{n = - \infty }^{\infty } L_{n} \left( z \right)\left[ {\frac{{2{\tilde{\Gamma }}}}{{\left( {\omega \mp n\tilde{\omega }_{0} - \tilde{\varepsilon }_{d} - \tilde{U}n_{d, - \sigma } } \right)^{2} + {\tilde{\Gamma }}^{2} }}} \right],$$

where $n$ is the number of phonons and ${\tilde{\Gamma }} = {\Gamma }_{0} e^{{ - \lambda^{2} \left( {2f_{ph} + 1} \right)}} .$ The analytical continuation rule of Langreth is now applied to the Dyson equations for the Keldysh Green functions to obtain:$\tilde{G}^{ > \left( < \right)} \left( \omega \right) = \tilde{G}_{dd}^{r} \left( \omega \right) {\tilde{\Sigma }}^{ > \left( < \right)} \left( \omega \right) \tilde{G}_{dd}^{a} \left( \omega \right),$ where ${\tilde{\Sigma }}^{ < } \left( \omega \right) = i {\tilde{\Gamma }}\left[ {f_{S} \left( \omega \right) + f_{D} \left( \omega \right)} \right] , {\tilde{\Sigma }}^{ > } \left( \omega \right) = - i {\tilde{\Gamma }} \left[ {2 - (f_{S} \left( \omega \right) + f_{D} \left( \omega \right))} \right].$ The Green functions $\tilde{G}_{dd}^{r,a} \left( \omega \right)$ are determined by making use of the equation of motion method and subsequently $\tilde{G}^{ > \left( < \right)} \left( \omega \right)$ and ${\text{A}}\left( \omega \right)$ are obtained and hence the tunneling current is finally obtained. To deal with the onsite repulsive coulomb correlation part of the system, we employ a mean-field approximation using the Hartree–Fock (HF) decoupling method. Therefore our results are well outside the Kondo regime.

### Differential conductance

The differential conductance $G$ is given by: $G = dJ/dV_{b}$. Straight-forward calculation gives

15 $$G = \frac{{e^{2} {\Gamma }}}{2h}\mathop \sum \limits_{n = - \infty }^{\infty } L_{ \pm n} \mathop \smallint \limits_{ - \infty }^{\infty } d\omega F_{n} \left( \omega \right)A\left( {\omega - n\tilde{\omega }_{0} } \right),$$

where

16 $$\begin{aligned} & F_{n} \left( \omega \right) = { }\frac{1}{{2k_{B} T}}\left[ {f_{s} \left( \omega \right)\left( {1 - f_{s} \left( \omega \right)} \right) + f_{D} \left( \omega \right)\left( {1 - f_{D} \left( \omega \right)} \right)} \right]\left[ {1 + \frac{1}{2} \left( {e^{{ - \frac{{n\tilde{\omega }_{0} }}{{k_{B} T}}}} - 1} \right)\left\{ {f_{S} \left( {\omega - n\tilde{\omega }_{0} } \right) + f_{D} \left( {\omega - n\tilde{\omega }_{0} } \right)} \right\}} \right] \\ & \quad \quad \quad \quad \quad + \frac{1}{{4k_{B} T}}\left( {e^{{ - \frac{{\left( {n\tilde{\omega }_{0} } \right)}}{{k_{B} T}}}} - 1} \right)\left( {f_{S} \left( \omega \right) - f_{D} \left( \omega \right)} \right) \times \left[ {f_{S} \left( {\omega - n\tilde{\omega }_{0} } \right)\left\{ {1 - f_{S} \left( {\omega - n\tilde{\omega }_{0} } \right)} \right\} - f_{D} \left( {\omega - n\tilde{\omega }_{0} } \right)\left\{ {1 - f_{D} \left( {\omega - n\tilde{\omega }_{0} } \right)} \right\}} \right] \\ \end{aligned}$$

## Results and discussions

In our numerical computation, the phonon energy $\hbar \omega_{0}$ is set as the scale of energy and the following values for the SMT parameters are considered (unless we specify otherwise): $\varepsilon_{d} = 0, eV_{g} = 0, \Gamma = 0.2, U = 3$ and $eV_{m} = 0.5.$ Chen et al.^25^ explained clearly that $eV_{m}$ is related to the chemical potentials of an SMT device and how it affects the line shape of the spectral density function. In $\lambda = 0$ case, the spectral function is symmetric with respect to $\tilde{\varepsilon }_{d}$ which implies $eV_{m} = \varepsilon_{d} = 0$ but, generally the spectral density function in the presence of e–p interaction is asymmetric with respect to $\tilde{\varepsilon }_{d}$ as $eV_{m} \ne \tilde{\varepsilon }_{d}$. So, we consider $eV_{m} \ne 0$ in our calculation. In order to examine the role of temperature on the transport properties of an SMT device, we investigate the behaviour of Spectral density function $A$, Current density $J$ and differential conductance $G$ with respect to SMT parameters for different values of temperature. We also study the behaviour of these quantities directly as a function of temperature.

**Figure 2:**
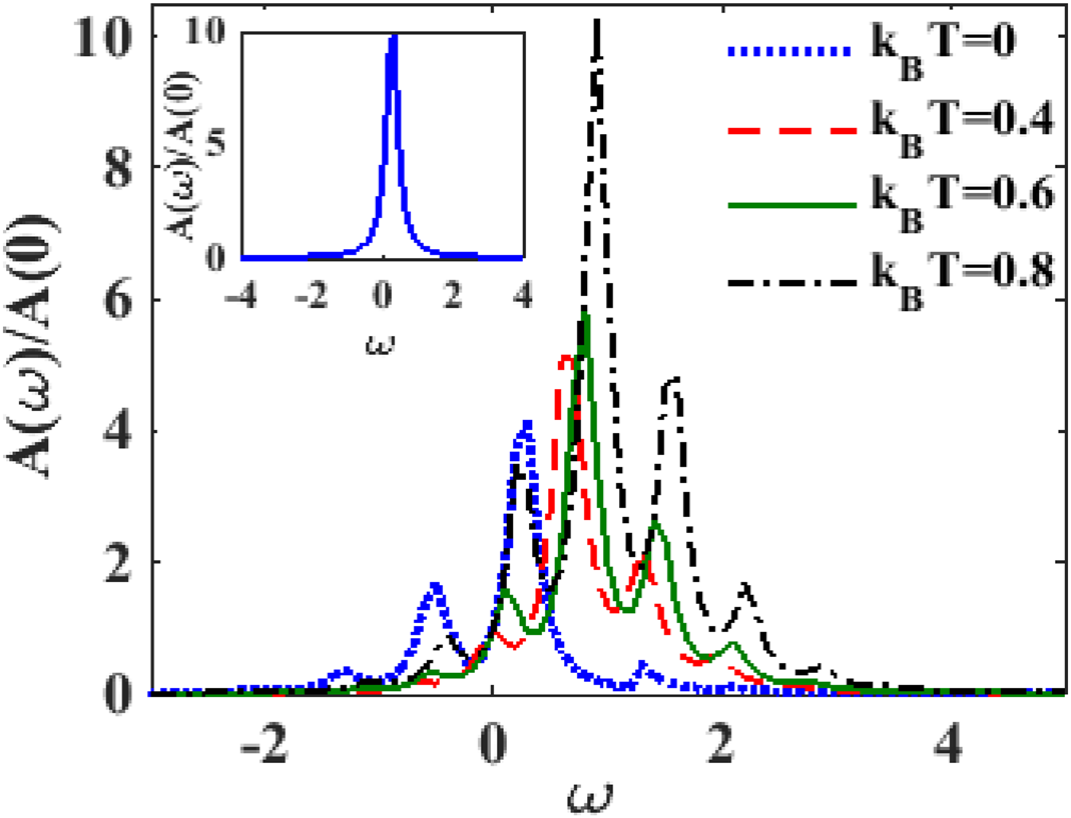
$A\left( \omega \right)/A\left( 0 \right)$ as a function of $\omega$ for different values of $k_{B} T$ at $\lambda = 0.6,\gamma = 0.02,eV_{b} = 2.5,eV_{m} = 0.5$.

**Figure 3:**
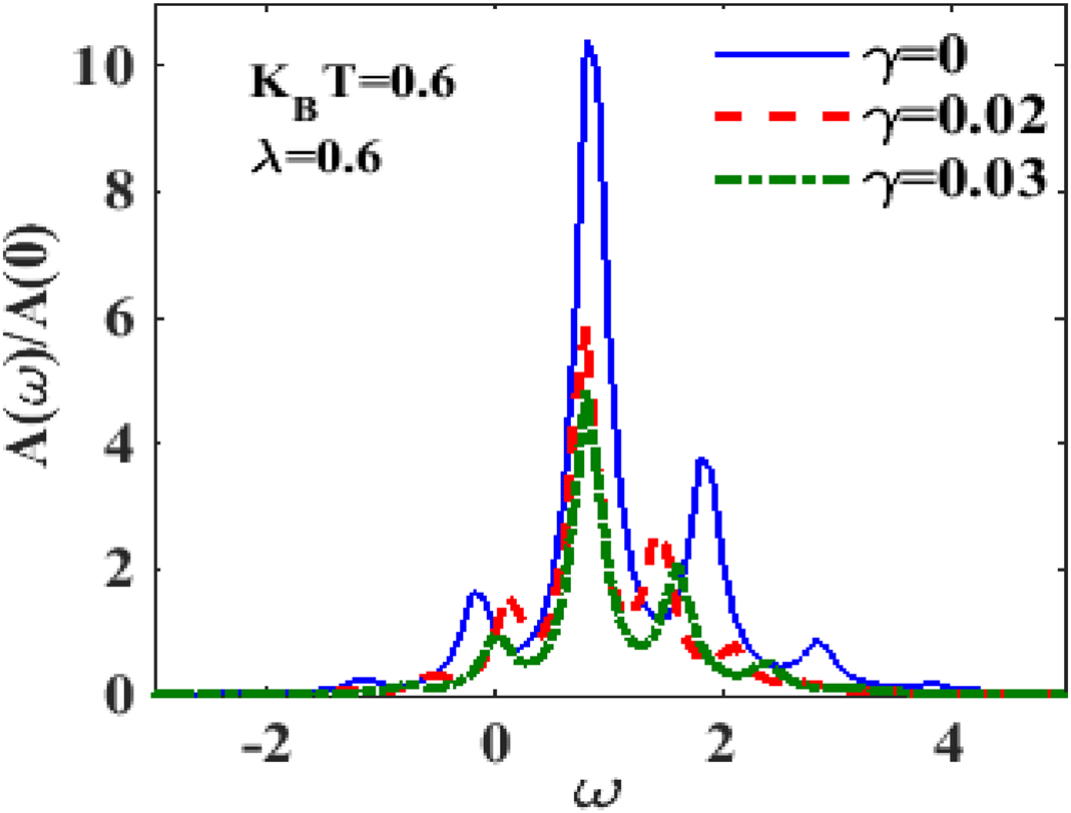
$A\left( \omega \right)/A\left( 0 \right)$ versus $\omega$ for different values of $\gamma$ at $\lambda = 0.6,k_{B} T = 0.6, eV_{b} = 2.5,eV_{m} = 0.5$.

In Fig. 2 we study the behaviour of the spectral function $A\left( \omega \right)$ with respect to energy $\omega$ at $\lambda = 0.6, eV_{m} = 0.5, eV_{b} = 2.5$. The inset shows the result for $\lambda = 0$ and $k_{B} T = 0$. The Lorentzian peak at zero resonant energy is clearly visible. The main plots in Fig. 2 show the behaviour of $A\left( \omega \right)$ as a function of energy $\omega$ for different values of temperature ($T$) in the presence of e–p interaction. Figure 2 also shows that at $T = 0,$ as the e–p interaction is switched on, the polaronic effect comes into play that causes renormalization of the SMT parameters and consequently the central peak in the spectral density undergoes a red shift and side peaks appear at: $\omega \mp n\tilde{\omega }_{0}$ in it. This was already shown in^26,27^. The phonon side bands correspond to the excitation of phonons. The tunneling of electrons into the QD and out of it can occur by emitting or absorbing a phonon which shows up in the spectral function in the form of side bands. Higher-order phonon processes are less likely to happen and so the heights of the side bands decrease as the energy increases. The scenario becomes more interesting at nonzero temperature. At $T \ne 0,$ the central peak becomes sharper, taller in height and undergoes a blue shift. Also the temperature has a diminishing effect on the phonon side bands in the negative energy region while in the positive energy region, the side bands tend to grow with temperature. In Fig. 3 we show the behavior of $A\left( \omega \right)$ as a function of $\omega$ for different values of the damping rate $\gamma$ at a certain finite temperature $\left( {k_{B} T = 0.6} \right)$ and finite e-p coupling constant ($\lambda = 0.6)$. One may notice that with increasing dissipation, the spectral function peak reduces in height and broadens in width. As discussed in ^26^, this implies that the role of dissipation is to reduce the occupancy of the phonon side bands.

**Figure 4:**
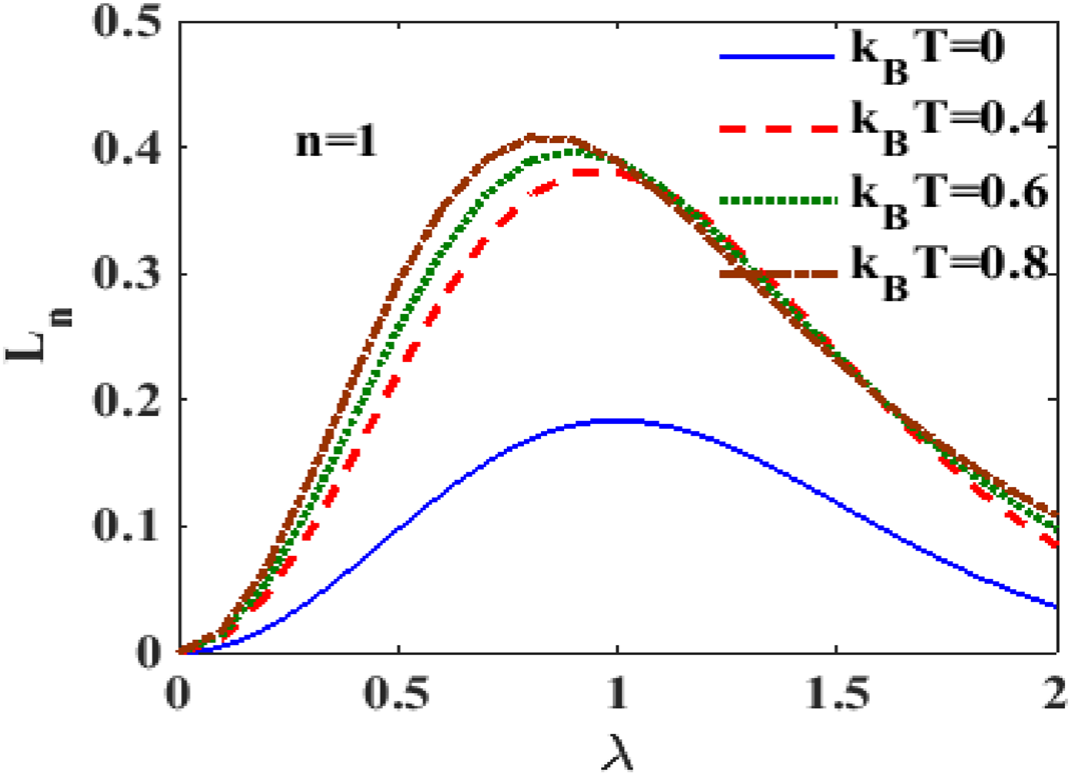
$L_{n}$ versus $\lambda$ for different $k_{B} T$ at $\gamma = 0.02$.

**Figure 5:**
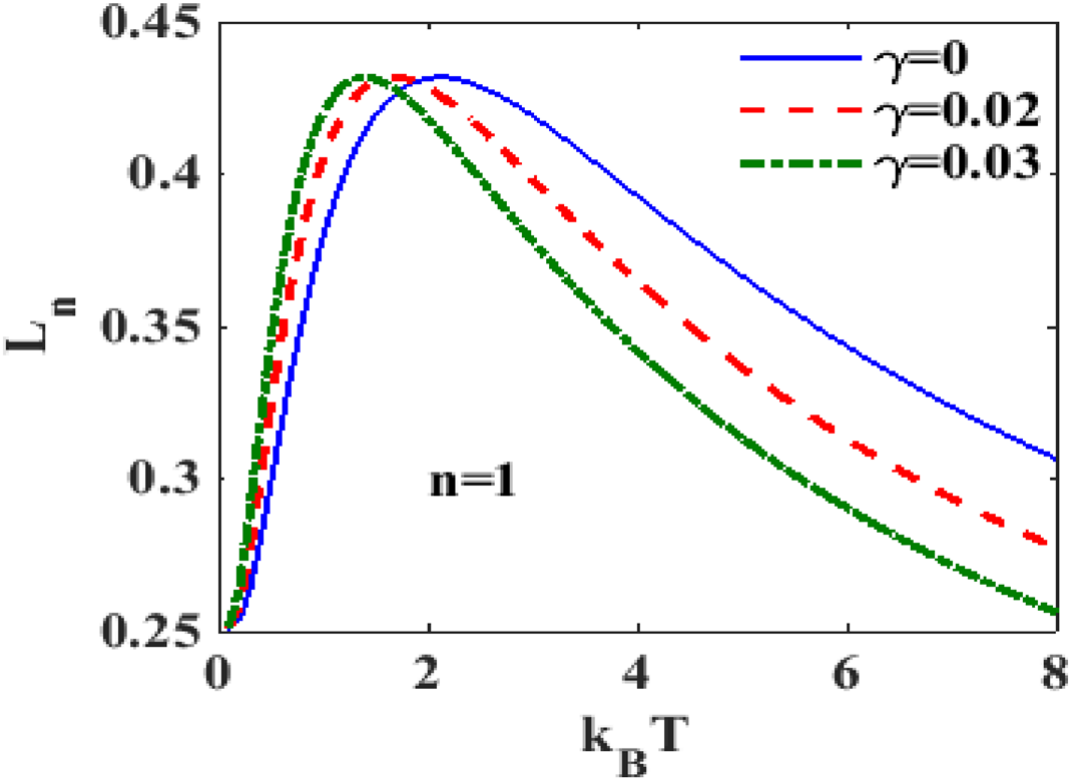
$L_{n}$ versus $k_{B} T$ for different $\gamma$ at $\lambda = 0.6$.

In^28^, the authors have shown the behavior of the spectral weight at zero temperature for different values of $n.$ To see the temperature dependence of the spectral weight, we plot in Fig. 4 the spectral weight versus e-p interaction strength $\lambda$ for different temperatures at $n = 1$. One can observe that the height of the spectral weight increases with increasing temperature $T$ up to a certain value of λ after which the temperature dependence of the spectral weight becomes marginal. With respect to λ, the spectral weight initially shows an increasing behaviour, then attains a maximum at a certain value of λ and finally decreases smoothly. We have also studied the behaviour of the spectral weight for higher values of $n$ and observed the same qualitative behavior as $n = 1$. But quantitatively, the value of the spectral weight undergoes significant reduction with increasing $n$. (We have not shown this here to save space). To see the effect of damping rate at $n = 1$, we plot in Fig. 5, the spectral weight $L_{1}$ with respect to $T$ for different values of damping rate. One can clearly see that that the spectral weight $L_{1}$ increases sharply with $T$ at low $T$, develops a maximum at a certain value of $T$ beyond which it deceases with further increase in $T.$ It is also visible that the spectral weight $L_{1}$ increases with dissipation up to a certain temperature beyond which it decreases with $T$.

**Figure 6:**
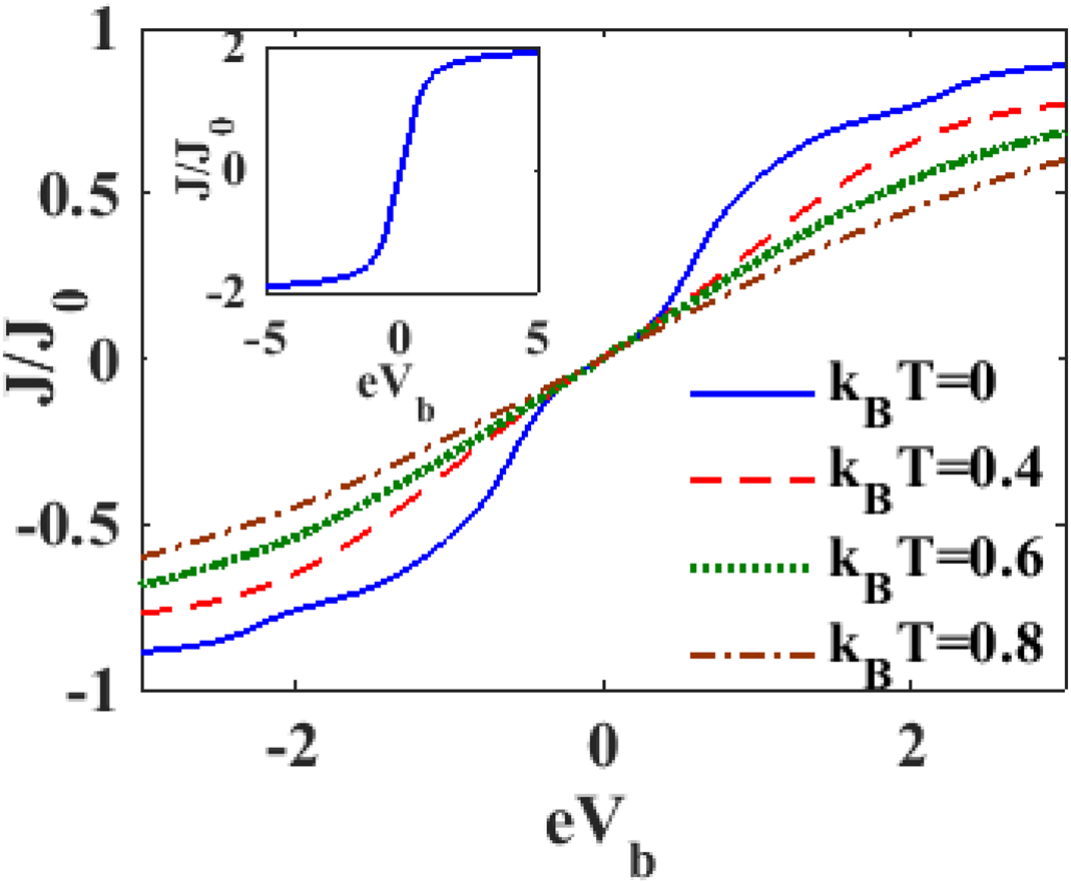
$J/J_{0}$ versus $eV_{b}$ for different values of $k_{B} T$ at $\lambda = 0.6,eV_{m} = 0.5,\gamma = 0.02$.

**Figure 7:**
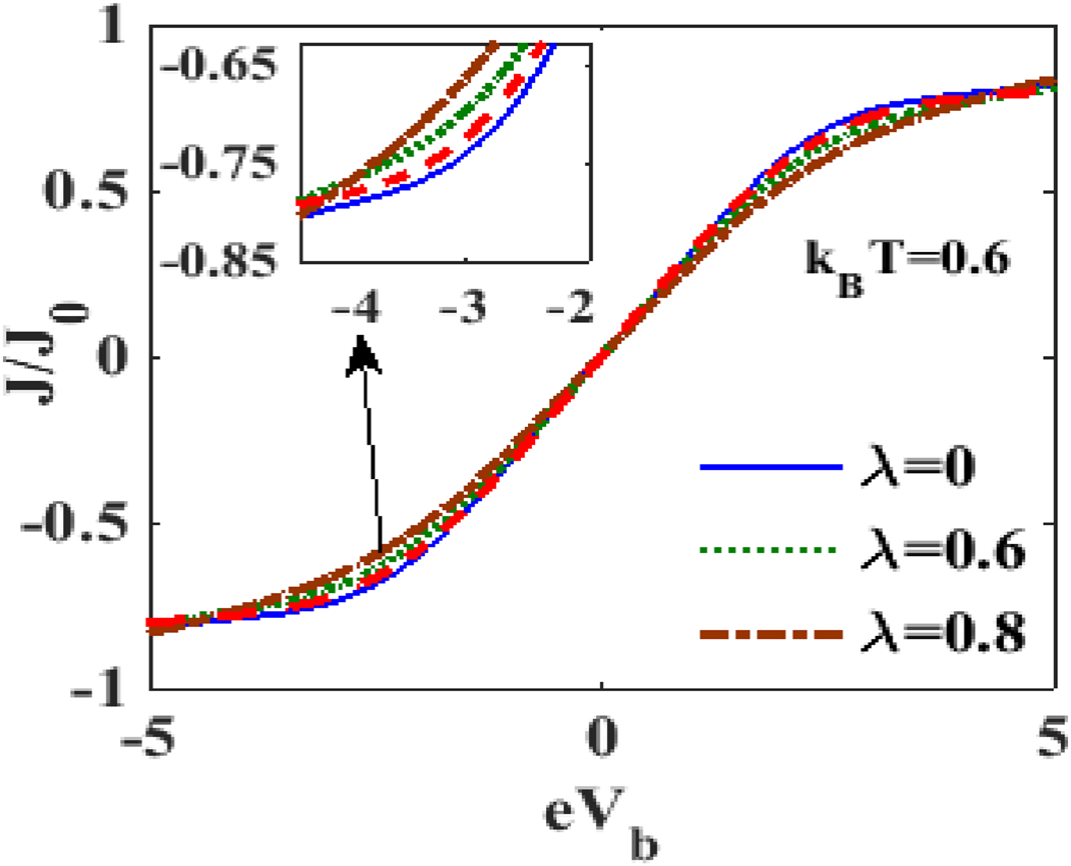
$J/J_{0}$ versus $eV_{b}$ for different values of $\lambda$ at $eV_{m} = 0.5,\gamma = 0.02,k_{B} T = 0.6$.

We measure the current density $J$ in units of $J_{0} = e{\Gamma }_{0} /2h$ which is the current density in the absence of e-p interaction. The variation of the normalized current density $J/J_{0}$ with the bias voltage $V_{B}$ at a particular e-p coupling constant $\lambda$ is displayed in Fig. 6 for different values of $T.$ The inset in the figure which presents the result for $\lambda = 0, T = 0,$ $\gamma = 0,$ suggests that the current density increases linearly with the bias voltage before it saturates. The main figure reveals that for a given set of SMT parameters, $J$ decreases with increasing $T.$ James et al.^39^ have carried out an experimental investigation to study the current through an SMT device with graphene based zinc-porphyrin as the central molecule and have observed that the current density increases with the bias voltage but decreases with increasing temperature. They have also calculated the current density for the system in the presence of e-e and e-p interactions using the rate equation approach and their theoretical predictions agree reasonably with their experimental results. We would like point out that our results for the current density in a single-QD SMT device as a function of the bias voltage at different temperatures also qualitatively agrees with the results of James et al.^39^. In Fig. 7, we present the variation $J$ with respect to $V_{B}$ for different values of $\lambda .$ At a particular finite temperature, the current density is reduced by the e-p interaction because of the formation of polarons. However, for the range of electron–phonon coupling considered in the present analysis, the reduction is only marginal.

**Figure 8:**
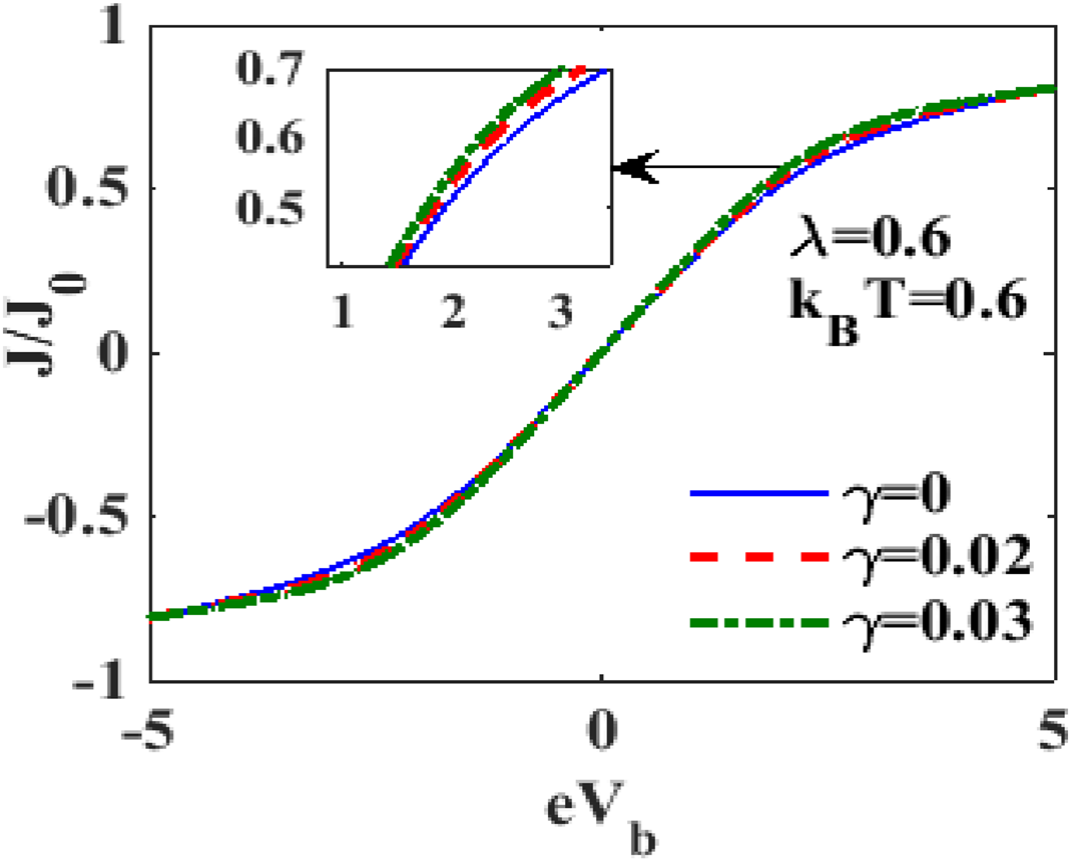
$J/J_{0}$ versus $eV_{b}$ for different values of $\gamma$ at $\lambda = 0.6,$ $eV_{m} = 0.5,k_{B} T = 0.6$.

To see the damping effect on the bias-voltage-dependance of current density, we plot in Fig. 8, $J$ with respect to $V_{B}$ for different values of the damping rate $\gamma$ with $\lambda = 0.6$ and $k_{B} T = 0.6$. $J$ increases only marginally with increasing $\gamma .$ We have also studied the variation of ${\text{J}}$ as a function of $eV_{b}$ for different values of ${\text{eV}}_{{\text{m}}}$ (not shown here) and observed that at low bias voltage, ${\text{V}}_{{\text{m}}} -$ dependence of ${\text{J}}$ is essentially insignificant while at higher bias voltage, ${\text{J}}$ shows a small but observable increase with ${\text{V}}_{{\text{m}}}$.

**Figure 9:**
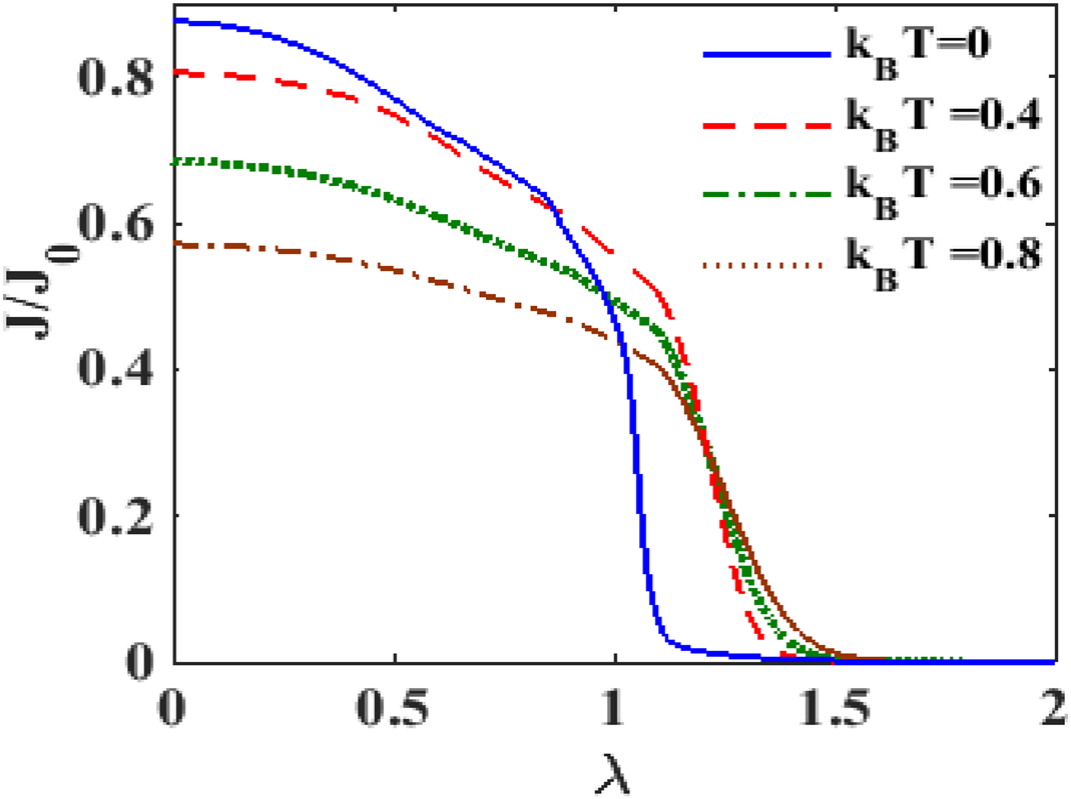
$J/J_{0}$ versus $\lambda$ for different values of $k_{B} T$ at $eV_{b} = 2.5,$ $eV_{m} = 0.5, \gamma = 0.02$.

**Figure 10:**
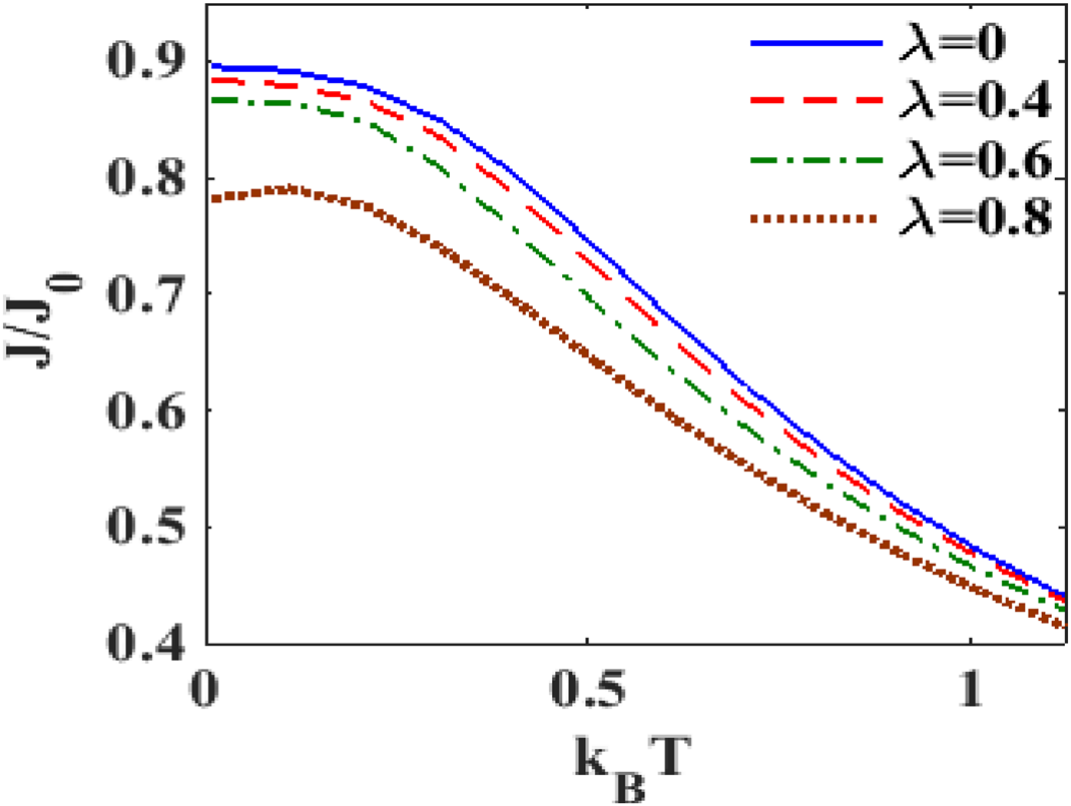
$J/J_{0}$ versus $k_{B} T$ for different values of $\lambda$ with $eV_{b} = 3.6,\gamma = 0.02, U = 3.$

Figure 9 shows directly the behavior of $J$ as a function of $\lambda$. It is clearly visible that the e-p interaction reduces the current density. Indeed the current density becomes zero at a certain value of $\lambda .$ The figure also shows that $J$ decreases as $T$ increases. Figure 10 shows directly the plot of $J$ versus $T$ for different values of $\lambda .$ One can clearly see that the current density decreases with increasing temperature. These results are in qualitative agreement with the results of James et al.^39^. Also these results are in conformity with Figs. 6 and 7. Since our results are for a simple model, we are not able to make a quantitative comparison with the results of James et al.^39^.

**Figure 11:**
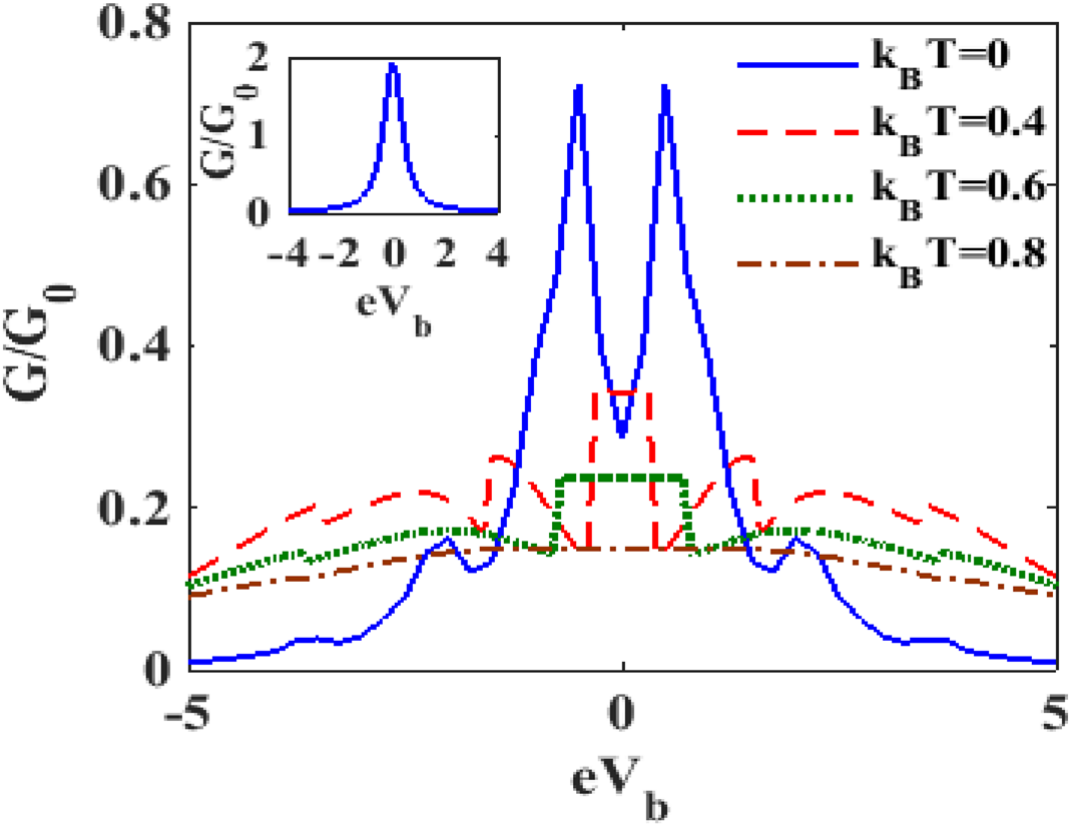
$G/G_{0}$ versus $eV_{b}$ for different values of $k_{B} T$ at $\lambda = 0.6, eV_{m} = 0.5, \gamma = 0.02$.

In Fig. 11 we present the variation of the normalized differential conductance $G/G_{0}$, with respect to the bias voltage $V_{B}$ for different values of temperature $T$, where $G_{0}$ is given by: $G_{0} = e^{2} {\Gamma }_{0} /2h$. The inset in Fig. 11 shows the plot at $T = 0K$ for $\lambda = 0$ and ${\upgamma } = 0.$ It is clear that in the absence of dissipation and the e-p interaction and at zero temperature $\left( {{\text{T}} = 0} \right)$, ${\text{G}}$ exhibits a single central peak that is symmetric in nature. The main figure shows that the e-p interaction splits the central peak into two symmetric peaks around λ = 0 and also gives rise to a few phonon side bands due to polaronic effect. One can observe that temperature in general reduces the differential conductance.

**Figure 12:**
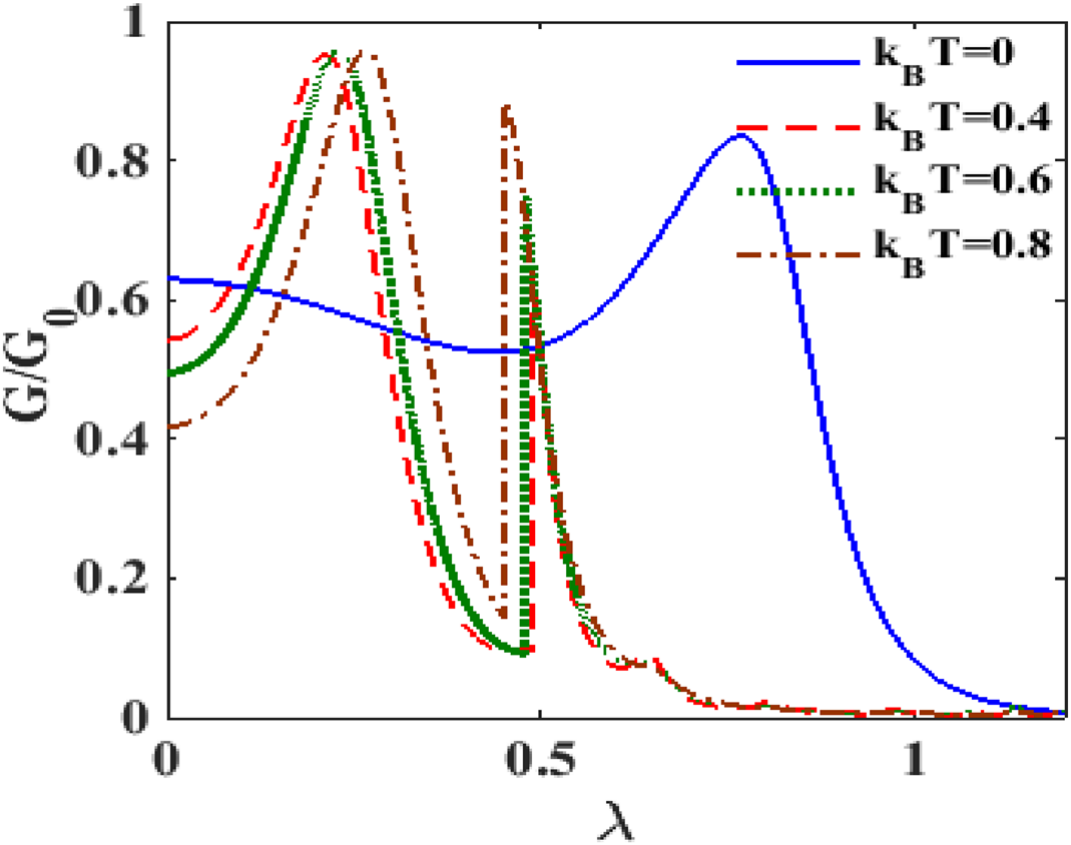
$G/G_{0}$ versus $\lambda$ for different values of $k_{B} T$ at $\lambda = 0.6, eV_{m} = 0.5, \gamma = 0.02$.

In Fig. 12 we plot the differential conductance ${\text{G}}$ as a function of e-p coupling constant $\lambda$ for different values of temperature $T$***. ***At ${\text{T}} = 0{\text{K}}$, as ${\uplambda }$ increases, ${\text{G }}$ initially decreases and then exhibits a shallow minimum. As ${\uplambda }$ increases further, ${\text{G}}$ develops a peak and finally reduces to zero. It is interesting to note that if the temperature is increased from zero, the behaviour of ${\text{G}}$ undergoes a qualitative change. To be more specific, at finite temperature, a double-peak structure is observed in the ${\text{G}}$ versus ${\uplambda }$ curves, one at (say) ${\uplambda }_{1}$ and the other one at (say) ${\uplambda }_{2}$, where ${\uplambda }_{2} > {\uplambda }_{1}$. The peak occurring at ${\uplambda }_{1}$ is much broader in width than the one occurring at ${\uplambda }_{2} .$ Furthermore, as ${\text{T}}$ increases, the peaks at ${\uplambda }_{1}$ shift towards higher values of ${\uplambda }$ while those at ${\uplambda }_{2}$ shift toward the lower values. With increasing ${\text{T}}$, the height of the second peak reduces whereas that of the first peak remains unaffected. At low values of ${\uplambda }$, one can clearly see that ${\text{G }}$ decreases with increasing temperature.

**Figure 13:**
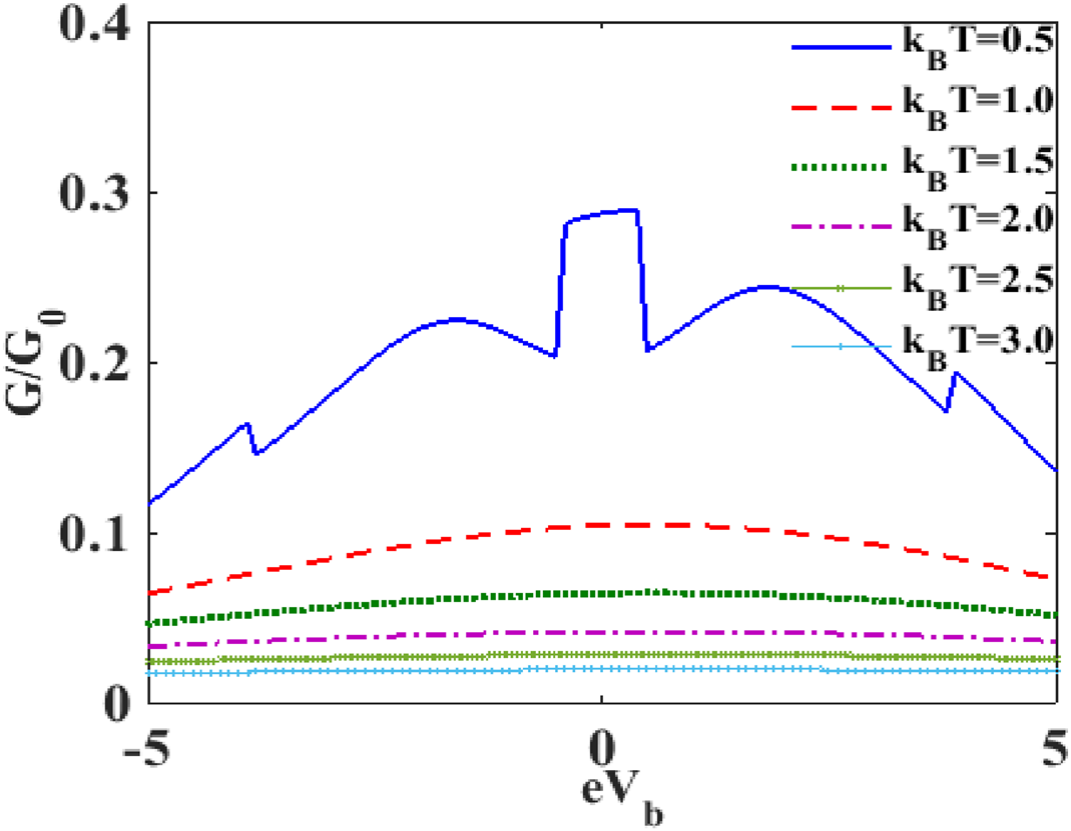
$G/G_{0}$ versus $eV_{b}$ for different values of $k_{B} T$ with ${\uplambda } = 0.6,{\text{ eV}}_{{\text{m}}} = 0.5,{{\gamma }} = 0.02$.

In Fig. 13, we plot ${\text{G}}$ as a function of $V_{B}$ for different values of $T$. We can clearly see that ${\text{G}}$ decreases with increasing temperature as shown in Fig. 1. However, we find that as temperature becomes high, the side bands completely disappear and the $G - eV_{b}$ curves exhibit only a single and broad maximum. As $T$ is further increased, the curves become essentially flat independent of $V_{b}$.

**Figure 14:**
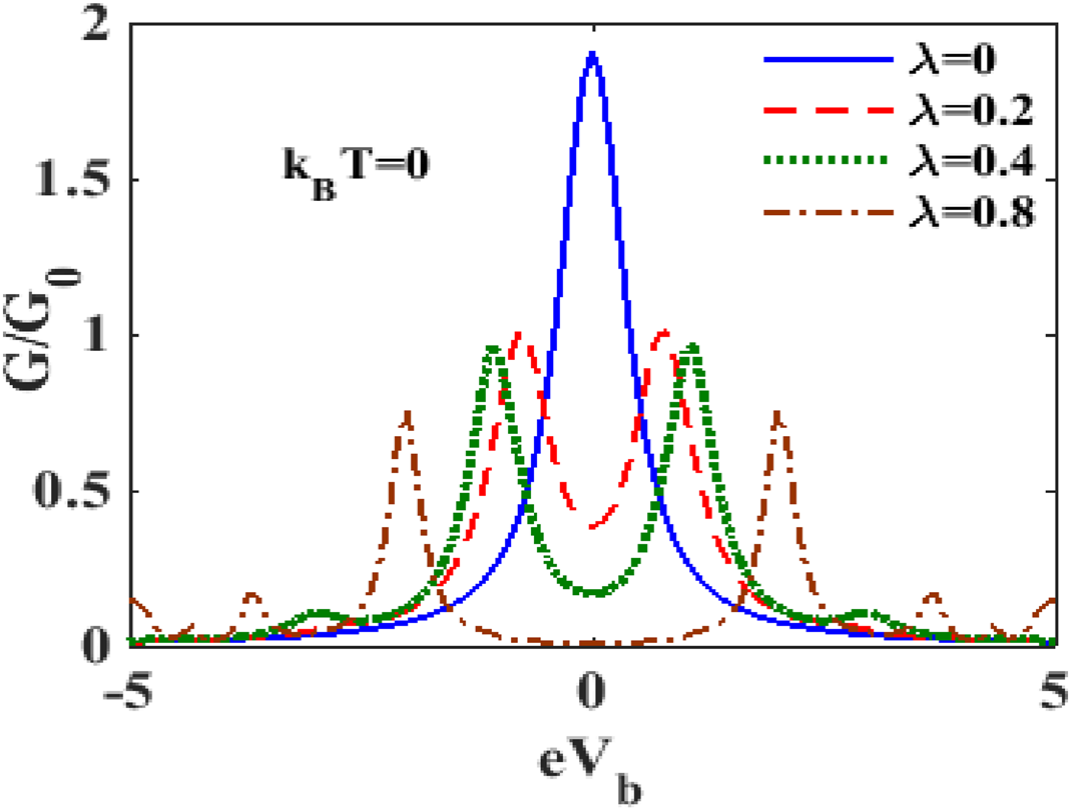
$G/G_{0}$ as a function of $eV_{b}$ for different values of $\lambda$ with ${\text{ eV}}_{{\text{m}}} = 0.5,{{\gamma }} = 0.02$ at $k_{B} T = 0$.

**Figure 15:**
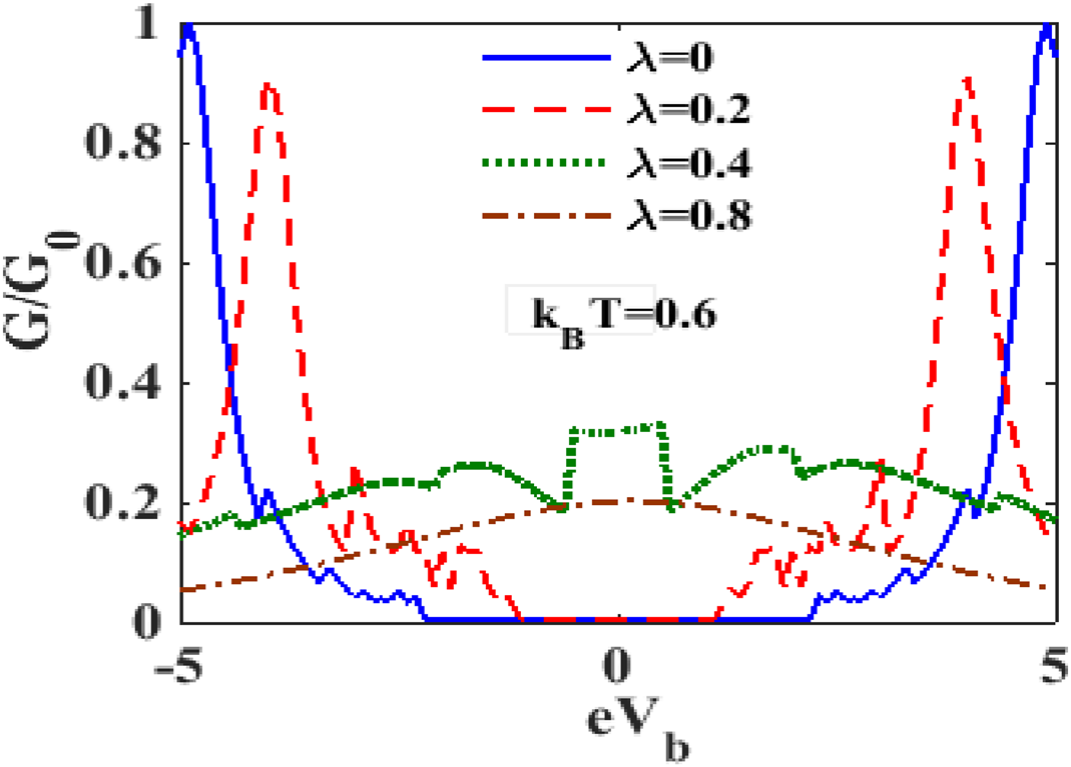
$G{/}G_{0}$ as a function of $eV_{b}$ for different values of $\lambda$ with ${\text{ eV}}_{{\text{m}}} = 0.5,{{\gamma }} = 0.02$ at $k_{B} T = 0.6$.

Figures 14 and 15 display the variation of ${\text{G}}$ with respect to $V_{B}$ for different values of $\lambda$ at $k_{B} T = 0$ and $k_{B} T = 0.6$ respectively. One can see that at zero temperature and in the absence of e-p interaction $G - V_{b}$ curve has a single central peak structure. Figure 14 also shows that in the presence of e-p interaction, the central peal is split into two peaks giving rise to a central minimum. As $\lambda$ increases, the peaks decrease in height and move away from each other, and the central minimum also moves down. At $\lambda = 0.8$, ${\text{G}}$ becomes zero for small values of $V_{b}$ on both sides of $V_{b} = 0$. Figure 15 shows that at finite temperature, even at $\lambda = 0$, the central peak of $G$ undergoes a splitting and ${\text{G}}$ remains zero over a range of values on either side of $V_{b} = 0$. As $\lambda$ is increased but is still kept low, the peaks decrease in height and come closer to each other and the window of $V_{b}$ values over which ${\text{G}} = 0$ decreases. Interestingly, if $\lambda$ is increased beyond a critical value, the sharp double peak structure disappears and a single broad maximum with the maximum at $V_{b} = 0$, appears. As $\lambda$ is further increased, an interesting structure with a flat maximum around $V_{b} = 0$ is observed.

**Figure 16:**
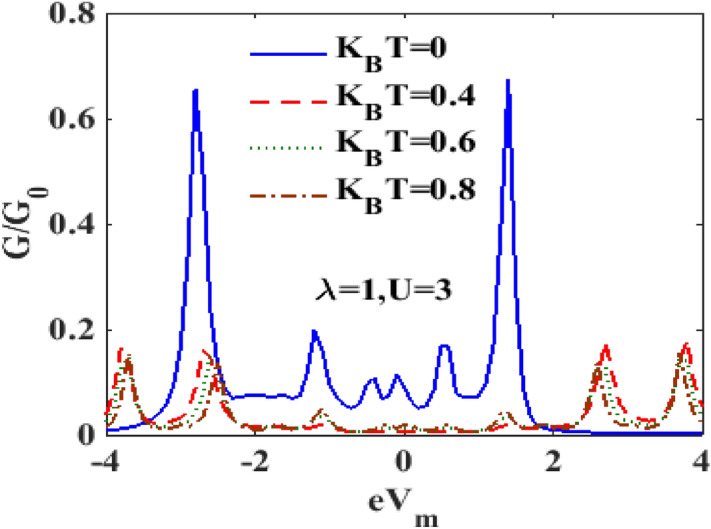
$G{/}G_{0}$ vs $eV_{m}$ for different values of $k_{B} T$ at ${ }eV_{b} = 3.6,\gamma = 0.02, U = 3 \;{\text{and}}\;\lambda = 1$.

**Figure 17:**
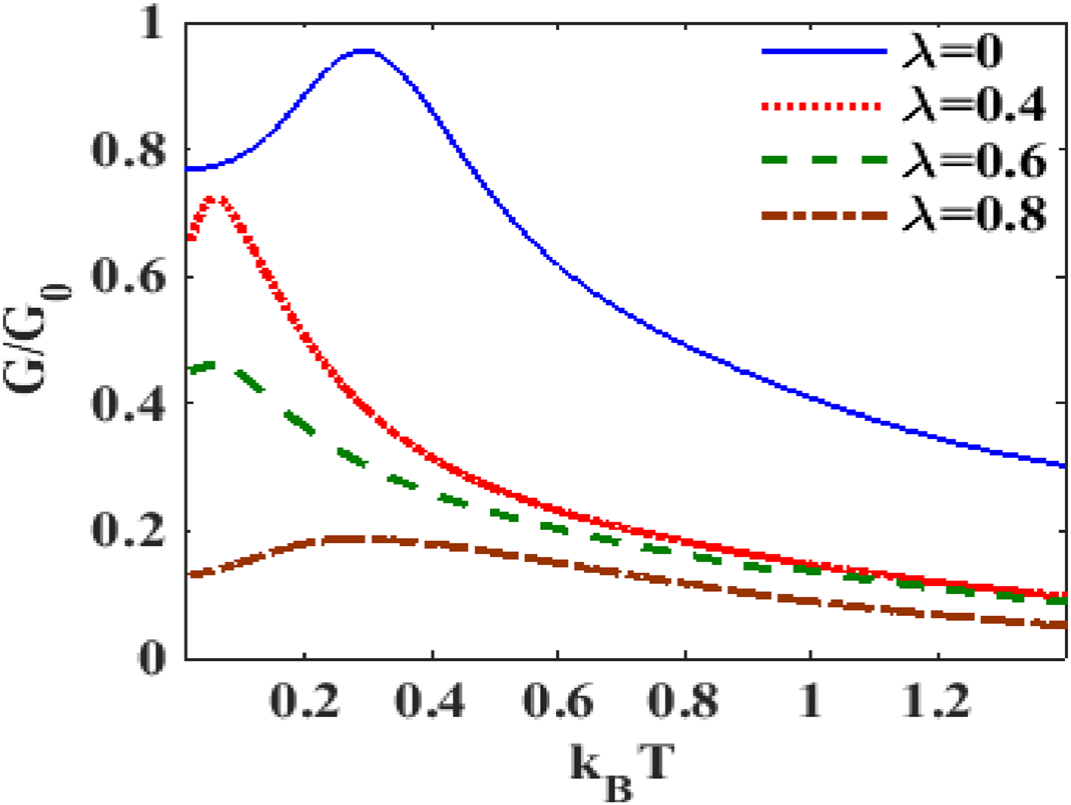
$G/G_{0}$ vs $k_{B} T$ for different values of $\lambda$ at $eV_{b} = 2.5,eV_{m} = 0.5,\gamma = 0.02$.

In Fig. 16, we show the behavior of $G$ with respect to $V_{m}$ in the presence of e-p and e-e interactions at different temperature. It is clear that, in general, the differential conductance together with its peak heights decreases with increasing temperature. One can also observe that the peaks shift towards the positive voltage with increasing temperature. At the mid-voltage range 0–1, a sudden drop in current density occurs (not shown here), the differential conductance is almost zero. As we have already pointed out, the peaks in the differential conductance represent the availability of energy levels for transport. At finite temperature, most of the peaks scale down. So the differential conductance reduces with increasing temperature. To examine the role of temperature on differential conductance directly, we plot in Fig. 17, $G$ versus $T$ for different values of $\lambda$. We observe that at $\lambda = 0.4$, $G$ initially increases with $T$, develops a peak at a cetain value of $T$ and then decreases smoothly with further increase in $T.$ With the increase in $\lambda$, the peak broadens and shifts towards to higher values of $T$. A critical study of the $G$ vs $T$ and $G$ vs $\lambda$ reveal that the variation of $G$ with respect to $T$ or $\lambda$ depends on the range of $T$ or $T$ under consideration.

**Figure 18:**
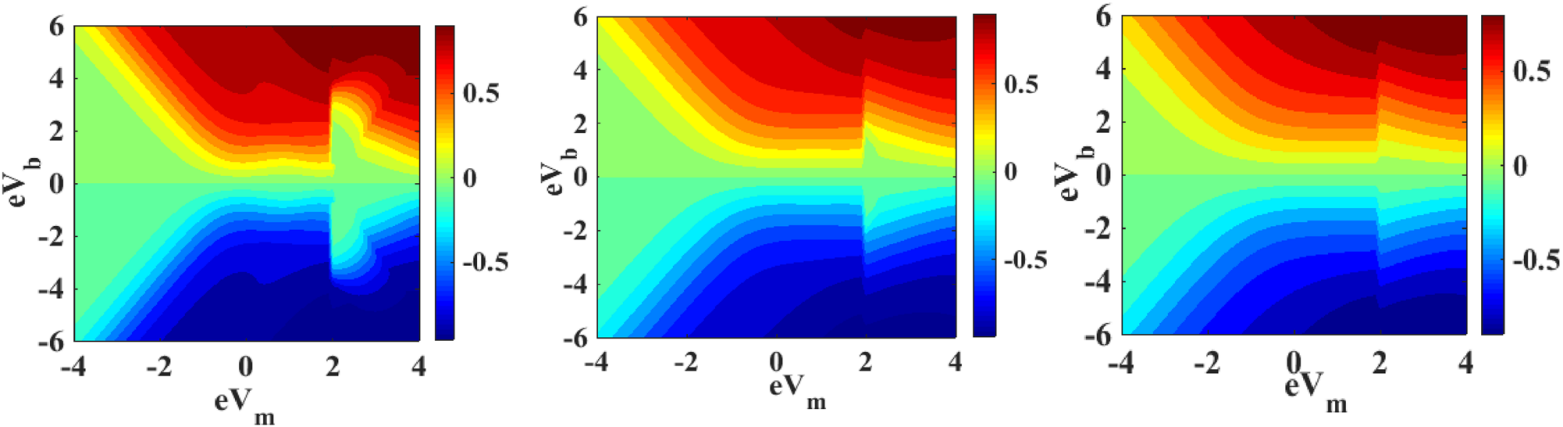
Map of $J$ in the V_b_ − V_m_**—**space for $\lambda = 0.6$ and $U = 3$ for (**a**) $k_{B} T = 0.4$; (**b**) $k_{B} T = 0.6$; (**c**) $k_{B} T = 0.8$.

**Figure 19:**
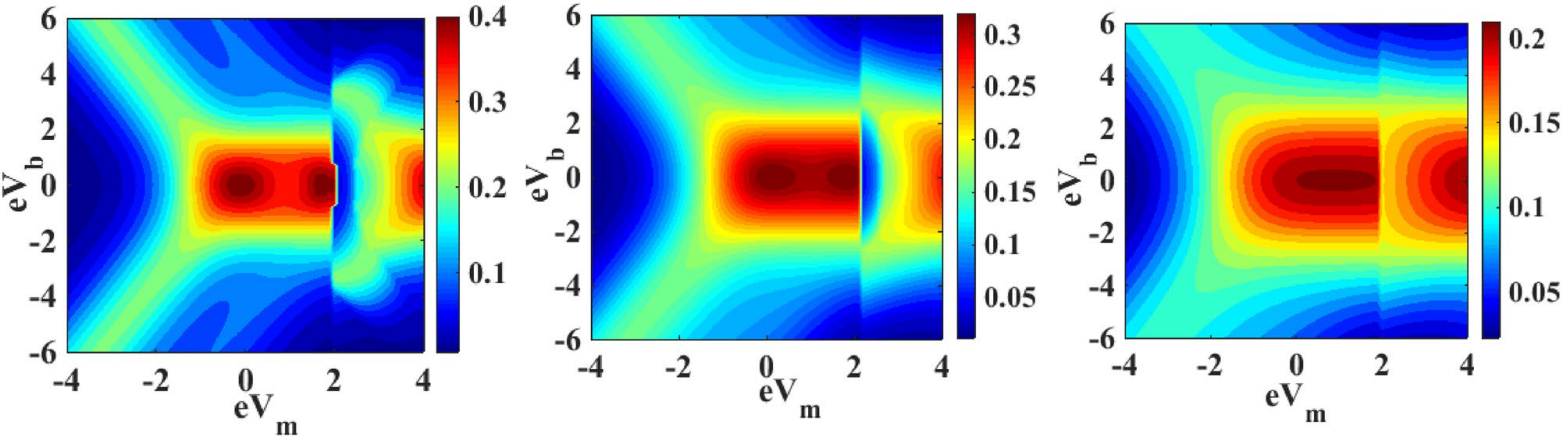
Map of $G$ in the V_b_ − V_m_**—**space for $\lambda = 0.6$ and $U = 3$ for (**a**) $k_{B} T = 0.4$; (**b**) $k_{B} T = 0.6$; (**c**)$k_{B} T = 0.8$.

We have already mentioned in Sect. 3.3 that the chemical potentials play an important role on the occupancy of the SMT level and on the phonon-assisted tunneling into the QD energy level. Since the chemical potentials are directly related to the bias voltage $V_{b}$ and mid-voltage $V_{m}$ of the system, it is important to study the dependence of the current density and differential conductance on $V_{b}$ and $V_{m}$. Figures 18 and 19 show the contour maps of the current density and the differential conductance respectively as a function of ${\text{eV}}_{{\text{b}}}$ and ${\text{eV}}_{{\text{m}}}$ in the presence of e–p and e–e interactions and quantum dissipation for different values of temperature. One may note from Fig. 18 that $J$ decreases with increasing temperature and also the boundary regions of the map shrink with increasing temperature. Similarly, Fig. 19 show that ${\text{G}}$ decreases with increasing temperature. However, the boundary regions in G-curves seem to broaden with temperature.

## Conclusion

We have investigated, in this work, the quantum dissipative effect on the electronic transport properties of a single molecular transistor at finite temperature in the presence of e-e and e-p interactions. The dissipative effect arises from the interaction of the QD phonon with the phonons of the substrate that plays the role of a heat reservoir. It has been assumed that the QD phonon interacts with the phonons of the substrate according to the Caldeira-Leggett model. This interaction gives rise to quantum dissipation and has been treated exactly by a canonical transformation which leads to the renormalization of the frequency of the QD phonon. The e-p interaction term has been decoupled by applying the standard Lang-Firsov transformation followed by an averaging with respect the zero-phonon state. Finally the transport parameters are determined by employing the Keldysh technique and the equation of motion method and the effect of temperature, damping rate and e-p interaction on the spectral function, current density and differential conductance are investigated. Also the quantum dissipative effects on the spectral weight are studied at finite temperature. It is observed that the spectral weight increases with increasing damping rate. It is also found that the damping enhances the current density at finite temperature but not as significantly as at zero temperature. As expected, the current density decreases with increasing e-p interaction and temperature. The differential conductance also behaves in the similar way. Our results for the current density qualitatively agree with their experimental results of James et al.^39^. This device has potential application as a spin filter in the presence of an external magnetic field or spin–orbit interactions.
